# The aspiration of precision glucocorticoid pharmacotherapy in immune-mediated diseases: where do we stand in integrating pharmacogenomics and biomarkers for individualized treatment?

**DOI:** 10.3389/fimmu.2026.1889058

**Published:** 2026-08-24

**Authors:** Matteo Colina, Gabriele Campana

**Affiliations:** 1 Rheumatology Service, Section of Internal Medicine - Department of Medicine and Oncology - Ospedale Santa Maria della Scaletta, Imola, Italy; 2 Alma Mater Studiorum, Department of Pharmacy and Biotechnology, University of Bologna, Bologna, Italy

**Keywords:** biomarkers, glucocorticoid receptor, glucocorticoid, immune-mediated diseases, immunomodulation, personalized therapy, pharmacogenomics, precision medicine

## Abstract

Glucocorticoids remain among the most widely prescribed immunosuppressive and anti-inflammatory agents worldwide, yet their clinical use is complicated by substantial inter-individual variability in both therapeutic response and adverse effects. Despite decades of clinical experience, glucocorticoid prescribing remains largely empirical, with limited integration of precision medicine approaches that could optimize therapeutic outcomes while minimizing toxicity. This review synthesizes current evidence on emerging precision approaches to glucocorticoid therapy across three key domains: pharmacogenomic determinants (including variants in NR3C1, FKBP5, CYP3A4/5, and ABCB1) that modulate immune cell responsiveness to glucocorticoids; pharmacokinetic variability and model-informed precision dosing strategies; and disease-specific biomarker-guided approaches across major immune-mediated conditions including respiratory diseases, rheumatologic conditions, inflammatory bowel disease, polymyalgia rheumatica, and critical illness. Genetic variants in glucocorticoid receptor signaling and drug metabolism pathways have suggested some associations with treatment response and adverse effects, although none are yet acknowledged as clinically actionable by consensus resources. Disease-specific immunological biomarkers including eosinophil counts, inflammatory cytokines, and pharmacodynamic endpoints suggest the promise of individualized dose optimization. Risk stratification for glucocorticoid-induced osteoporosis—mediated through OPG/RANKL signaling and monocyte-osteoclast crosstalk—incorporating both clinical factors and pharmacogenetic markers may facilitate targeted prevention strategies. The integration of pharmacogenomic testing, immune biomarker monitoring, and pharmacokinetic modeling represents an aspirational paradigm shift from empirical dosing to individualized immunomodulatory therapy. It should be acknowledged that, as of the time of writing, this precision framework remains largely investigational: routine clinical practice continues to rely predominantly on indication-specific regimens and empirical tapering protocols. This review is explicitly forward-looking, aiming to outline a research agenda for precision glucocorticoid therapy in immune-mediated diseases.

## Introduction

Glucocorticoids (GCs) are among the most widely prescribed drug classes in clinical medicine, with applications spanning rheumatology, pulmonology, gastroenterology, dermatology, and oncology (1, 2). Despite seven decades of clinical use since their introduction in 1948, glucocorticoid therapy remains largely empirical, relying on standardized weight-based dosing protocols that fail to account for substantial inter-individual variability in drug response (3).

The clinical challenge of glucocorticoid therapy is defined by a narrow therapeutic index and marked heterogeneity in both efficacy and toxicity. Some patients achieve complete disease remission with minimal adverse effects, while others experience treatment failure or develop serious complications including diabetes, osteoporosis, cardiovascular disease, and opportunistic infections. This variability reflects complex interactions between pharmacokinetic factors (drug absorption, distribution, metabolism, and elimination) and pharmacodynamic factors (receptor sensitivity, tissue responsiveness, and downstream signaling) (4, 5).

The genomics revolution has revealed that genetic polymorphisms account for 20%–95% of variability in drug response across therapeutic classes (6). For glucocorticoids specifically, variants in genes encoding the glucocorticoid receptor (NR3C1), drug transporters (ABCB1), and metabolizing enzymes (CYP3A4/5) significantly influence therapeutic outcomes (7, 8). Beyond genetics, disease-specific biomarkers—including inflammatory cytokines, autoantibodies, and metabolic markers—enable real-time monitoring of treatment response and early detection of adverse effects (9).

**Figure 1:**
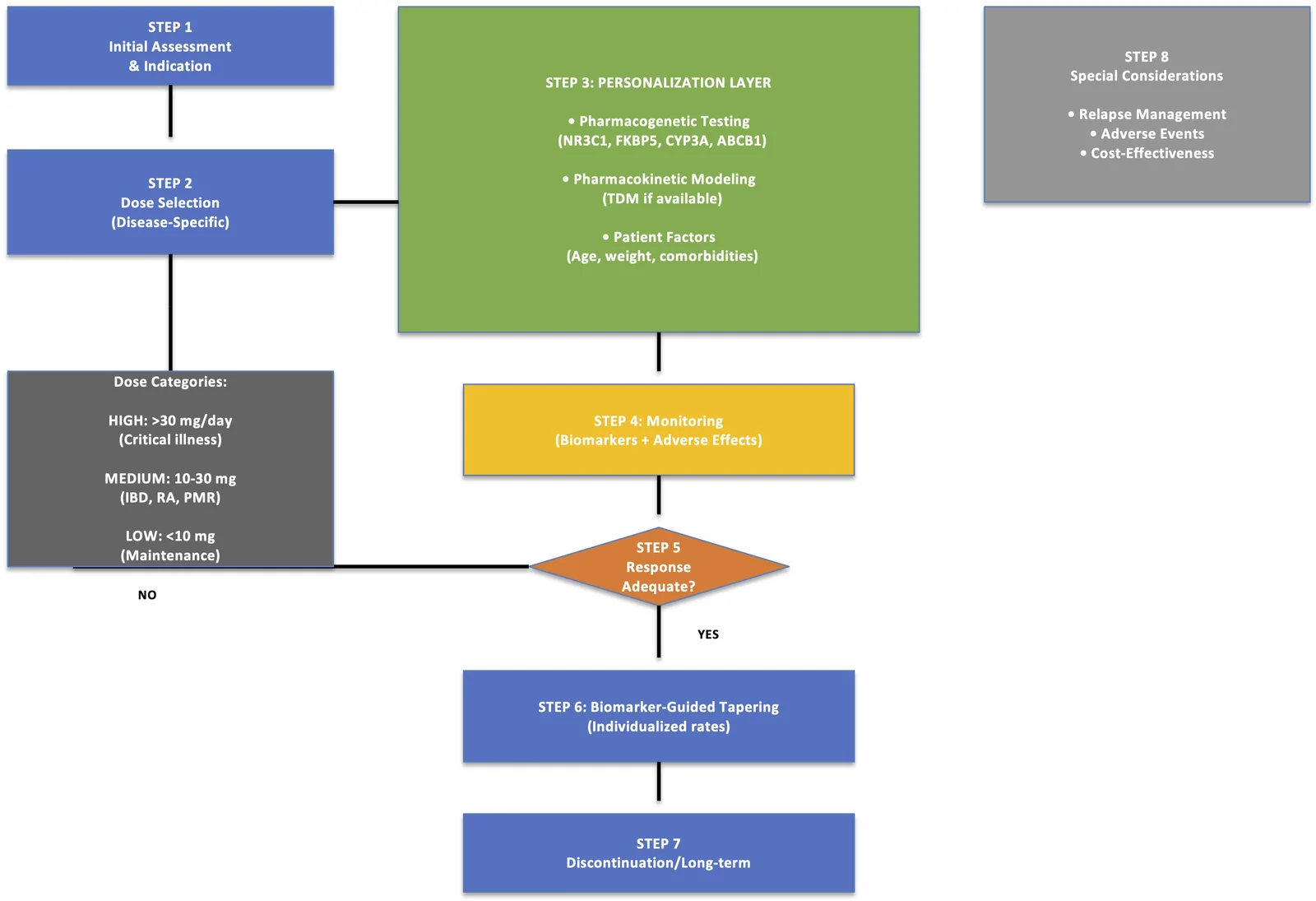
Clinical decision algorithm for precision glucocorticoid therapy. Comprehensive decision algorithm integrating pharmacogenomic testing, pharmacokinetic modeling, and biomarker-guided monitoring to individualize glucocorticoid dosing and tapering strategies (**Figure 2**). The algorithm proceeds through eight sequential steps: 1) initial clinical assessment and indication evaluation; 2) disease-specific dose selection based on published protocols; 3) personalization layer incorporating pharmacogenetic testing (NR3C1, FKBP5, CYP3A4/5, and ABCB1 variants), pharmacokinetic modeling where therapeutic drug monitoring is available, and patient-specific factors (age, weight, comorbidities, and concomitant medications); 4) biomarker and adverse effect monitoring using disease-appropriate markers; 5) response adequacy assessment incorporating clinical and biomarker endpoints; 6) biomarker-guided tapering with individualized reduction rates; 7) discontinuation planning or transition to long-term maintenance therapy; and 8) special considerations including relapse management, adverse event mitigation, and cost-effectiveness optimization. The algorithm incorporates a feedback loop returning to dose adjustment (Step 2) when treatment response is inadequate. Color coding indicates main clinical steps (blue), decision points (orange), personalization approaches (green), and monitoring activities (yellow). Dose categories (high >30 mg/day, medium 10–30 mg/day, and low <10 mg/day prednisone equivalent) are referenced to guide initial selection and risk stratification.

Furthermore, the clinical imperative for precision glucocorticoid therapy extends beyond dose optimization in chronic disease: it encompasses the critical challenge of glucocorticoid dependence—both physiological (through hypothalamic–pituitary–adrenal (HPA) axis suppression) and functional (through symptom recurrence during tapering despite the absence of active disease). The landmark COBRA trial demonstrated that high-dose step-down prednisolone combined with conventional disease-modifying antirheumatic drugs (DMARDs) produced superior radiographic outcomes in early rheumatoid arthritis, yet the optimal strategy for safely individualizing and terminating glucocorticoid exposure in clinical remission remains undefined (10). Many patients who could theoretically discontinue glucocorticoids based on disease activity indices such as Disease Activity Score in 28 joints (DAS28) fail to do so in practice, underscoring that the goal of reducing glucocorticoid exposure—universally shared—requires precision tools that current empirical approaches cannot provide (**Figure 1**).

**Figure 2:**
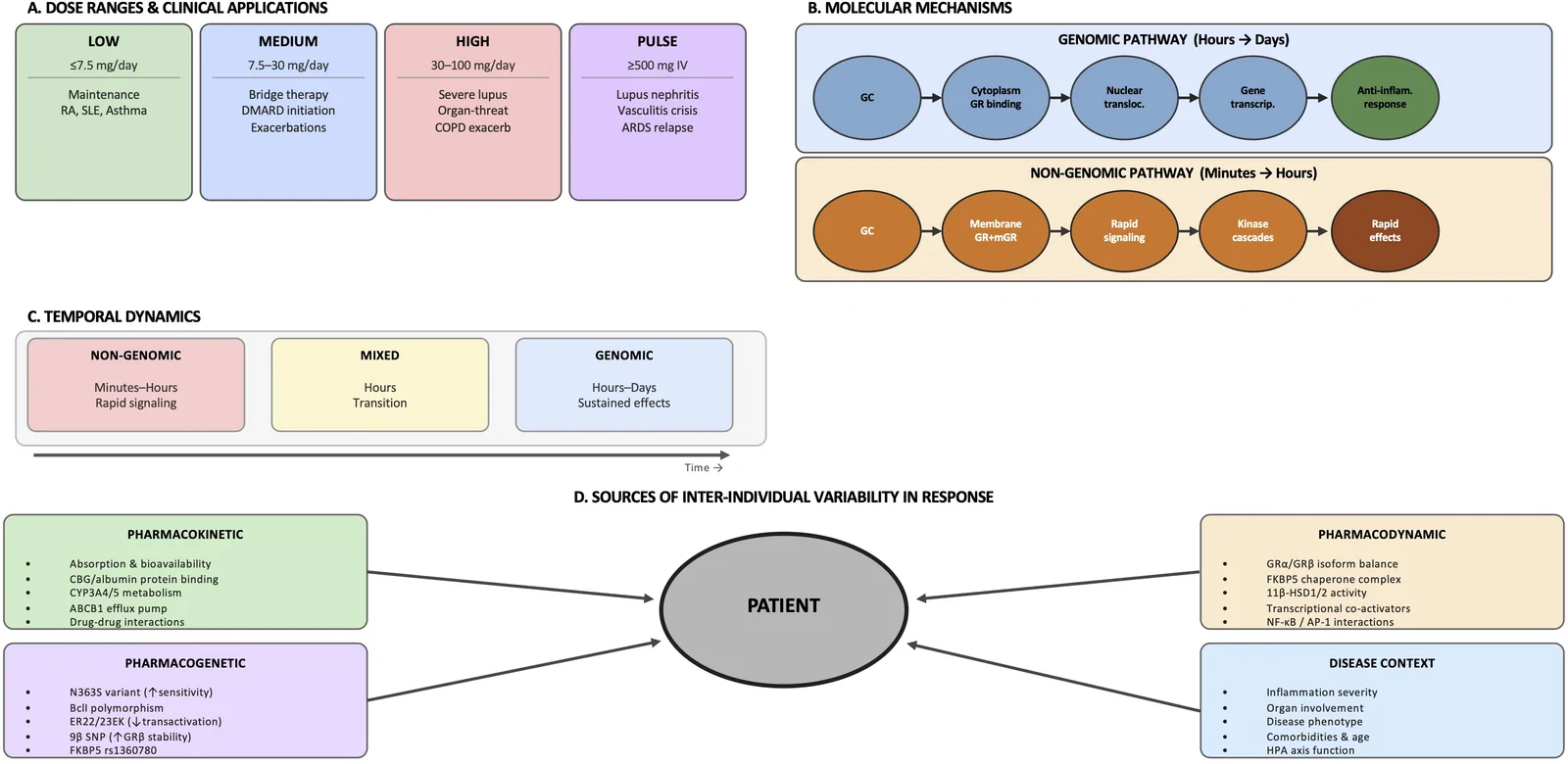
Framework for precision glucocorticoid therapy. **(A)** Sources of variability in glucocorticoid response. **(B)** Integration of pharmacogenomic and biomarker data. **(C)** Clinical decision algorithm. **(D)** Implementation workflow.

It should be acknowledged that, as of the time of writing, pharmacogenomic- and biomarker-guided glucocorticoid dosing remains an emerging aspirational framework rather than an established standard of care: routine clinical practice continues to rely predominantly on indication-specific regimens, clinical response assessment, comorbidity profiling, and empirical tapering protocols. This review is explicitly forward-looking, aiming to synthesize proof-of-concept evidence and outline a research agenda for the precision medicine approach to glucocorticoid therapy in immune-mediated diseases. , 

Precision medicine, defined as tailoring medical treatment to individual patient characteristics, offers a solution to the limitations of one-size-fits-all glucocorticoid dosing (11). This approach integrates multiple data streams: genetic testing to identify high-risk variants, biomarker monitoring to track disease activity and drug effects, pharmacokinetic modeling to predict optimal dosing, and clinical algorithms to guide treatment decisions. The goal is to maximize therapeutic benefit while minimizing toxicity through individualized dose selection, monitoring strategies, and adjunctive therapies (12, 13).

The economic implications are substantial. Glucocorticoid-related complications account for billions in healthcare costs annually (14). Adverse events requiring hospitalization, medication costs for managing side effects, and productivity losses from inadequate disease control create a compelling financial rationale for precision approaches. Pharmacoeconomic analyses suggest that biomarker-guided therapy may reduce overall costs through improved treatment targeting and reduced complications (15).

Implementation of precision glucocorticoid therapy requires integration across multiple domains: laboratory infrastructure for genetic testing and biomarker measurement, clinical decision support systems to interpret complex data, provider education on pharmacogenomic principles, and institutional protocols for systematic implementation. While challenges exist—including cost, reimbursement, and workflow integration—the evidence base supporting personalized approaches continues to strengthen (16).

This review synthesizes current evidence on precision glucocorticoid pharmacotherapy, examining: 1) mechanisms of glucocorticoid action and variability, 2) pharmacogenomic determinants of drug response, 3) disease-specific biomarkers for treatment individualization, 4) clinical applications across therapeutic areas, and 5) practical implementation strategies. We propose a framework for integrating these elements into clinical practice, with emphasis on actionable approaches that can be adopted in diverse healthcare settings.

The transition from empirical to precision-based glucocorticoid therapy represents a fundamental shift in treatment philosophy—from reactive management of complications to proactive optimization of individual patient outcomes. As our understanding of pharmacogenomic and biomarker determinants deepens, and as implementation tools become more accessible, precision glucocorticoid therapy is poised to become the standard of care across specialties.

## Mechanism of glucocorticoid action and sources of variability

### Glucocorticoid receptor structure and function

Glucocorticoids exert their effects primarily through the glucocorticoid receptor (GR; encoded by NR3C1), a ligand-activated transcription factor belonging to the nuclear hormone receptor superfamily (1, 7). The receptor consists of three functional domains: an N-terminal transactivation domain (NTD) responsible for gene activation, a central DNA-binding domain (DBD) containing two zinc fingers that recognize glucocorticoid response elements (GREs), and a C-terminal ligand-binding domain (LBD) that binds glucocorticoid molecules and contains a second transactivation function.

In the absence of ligand, GR resides in the cytoplasm as part of a multiprotein complex including heat shock proteins (HSP90 and HSP70), immunophilins (FKBP51 and FKBP52), and other chaperones that maintain receptor competence. Upon glucocorticoid binding, conformational changes trigger dissociation from the chaperone complex, receptor dimerization, nuclear translocation via importin proteins, and binding to GREs in target gene promoters (17).

The molecular mechanisms of GR action are diverse and context-dependent. Transactivation involves GR binding to positive GREs, recruiting coactivator complexes (including histone acetyltransferases and chromatin remodeling factors), and enhancing the transcription of anti-inflammatory genes such as DUSP1 (encoding MKP-1), TSC22D3 (GILZ), and ANXA1 (annexin A1) (7, 18). Transrepression, which accounts for many anti-inflammatory effects, occurs through multiple mechanisms: direct protein–protein interactions with pro-inflammatory transcription factors (NF-κB and AP-1), competition for limiting coactivators, recruitment of corepressors and histone deacetylases, and binding to negative GREs (7, 8).

Beyond classical genomic mechanisms, glucocorticoids trigger rapid non-genomic effects through membrane-associated receptors and cytoplasmic signaling pathways. These include the modulation of calcium flux, the activation of protein kinases (Src and PI3K), and direct effects on mitochondrial function. The relative contribution of genomic versus non-genomic mechanisms varies by tissue, dose, and temporal dynamics (19) (**Table 1**).

**Table 1:** Glucocorticoid dose ranges and predominant mechanism of action.

Dose category	Prednisone equivalent (mg/day)	Predominant mechanism	Clinical applications	Onset of action
Low dose	≤7.5	Genomic (transactivation/transrepression)	Maintenance therapy (RA, SLE, PMR, asthma); bridge to DMARDs	Hours to days
Medium dose	7.5–30	Mixed genomic + non-genomic	Disease flares, COPD exacerbations, moderate IBD activity, temporal arteritis	Hours
High dose	30–100	Predominantly non-genomic	Severe disease (active lupus, ARDS, asthma exacerbation, organ-threatening vasculitis)	Minutes to hours
Pulse dose	≥500 IV (methylprednisolone)	Non-genomic (rapid membrane effects)	Lupus nephritis, CNS vasculitis, ARDS, severe myositis, organ transplant rejection	Minutes

RA, rheumatoid arthritis; SLE, systemic lupus erythematosus; PMR, polymyalgia rheumatica; COPD, chronic obstructive pulmonary disease; IBD, inflammatory bowel disease; ARDS, acute respiratory distress syndrome; CNS, central nervous system; SNPs, single nucleotide polymorphisms.

### Pharmacokinetic variability

Glucocorticoid pharmacokinetics exhibit substantial inter-individual variation at every stage of drug disposition (6). The absorption of oral glucocorticoids is generally rapid and complete, with peak concentrations occurring within 1–2 h for most preparations. However, food effects, gastric pH, and intestinal transporter expression can influence bioavailability (1). Prednisone requires hepatic conversion to the active metabolite prednisolone via 11β-hydroxysteroid dehydrogenase type 1 (11β-HSD1), introducing an additional source of variability (20, 21).

Distribution is influenced by plasma protein binding—primarily to corticosteroid-binding globulin (CBG) and albumin—which varies with inflammation, pregnancy, and genetic polymorphisms in the SERPINA6 gene encoding CBG (22, 23). Only unbound glucocorticoid is pharmacologically active and available for tissue distribution. The volume of distribution varies twofold to threefold between individuals, affecting both peak concentrations and the duration of action (21).

Metabolism represents the most significant source of pharmacokinetic variability. Cytochromes P450 3A4 and 3A5 mediate 6β-hydroxylation of glucocorticoids, the primary metabolic pathway for most synthetic preparations (24, 25). CYP3A activity varies more than 20-fold in the population due to genetic polymorphisms, drug interactions, and environmental factors (26). Inducers (rifampin, phenytoin, and carbamazepine) can increase glucocorticoid clearance by twofold to fourfold, necessitating dose increases (21, 27). Inhibitors (ketoconazole, ritonavir, and grapefruit juice) reduce clearance and increase exposure, raising toxicity risk (27).

Additional metabolic pathways contribute to variability. 11β-HSD type 2 inactivates glucocorticoids through conversion to 11-keto metabolites, protecting mineralocorticoid receptors in the kidney (20). Polymorphisms affecting 11β-HSD2 expression influence both efficacy and mineralocorticoid side effects. Conjugation reactions (glucuronidation and sulfation) facilitate renal elimination and exhibit genetic variability in enzyme expression (20, 27).

Elimination kinetics vary with dose, formulation, and individual clearance capacity. Most glucocorticoids exhibit linear pharmacokinetics at therapeutic doses, with half-lives ranging from 2–4 h (hydrocortisone and prednisolone) to 36–54 h (dexamethasone and betamethasone). However, the saturation of metabolic pathways can occur at high doses, leading to non-linear kinetics and prolonged exposure (28).

### Pharmacodynamic variability

Pharmacodynamic variability reflects differences in tissue responsiveness to glucocorticoids independent of drug concentrations (6). Glucocorticoid sensitivity varies more than 100-fold between individuals, ranging from exquisite responsiveness to complete resistance. This variability arises from multiple mechanisms affecting receptor expression, function, and downstream signaling (6, 29).

Glucocorticoid receptor polymorphisms constitute a major determinant of sensitivity (30). The ER22/23EK variant (rs6189/rs6190), present in 2%–4% of Caucasians, reduces receptor transactivation activity and is associated with relative glucocorticoid resistance, requiring higher doses for therapeutic effect (31, 32). Conversely, the N363S polymorphism (rs6195), found in 3%–5% of the population, enhances receptor sensitivity and increases susceptibility to metabolic side effects. The BclI restriction fragment length polymorphism (rs41423247), affecting a glucocorticoid response element in the NR3C1 gene, influences receptor expression levels and clinical responsiveness (31, 33) (**Table 2**).

**Table 2:** Pharmacogenetic variants affecting glucocorticoid response.

Gene	Variant	Effect on GC response	Clinical implication	Evidence level
GR (NR3C1)	N363S	Increased sensitivity	Lower doses may suffice; ↑ metabolic side effects	Moderate
GR (NR3C1)	BclI	Increased sensitivity	Risk stratification for adverse effects	Moderate
GR (NR3C1)	ER22/23EK	Decreased sensitivity	May require higher doses; protective for metabolic effects	Limited
FKBP5	rs1360780	Altered sensitivity	Context-dependent (stress, psychiatric)	Moderate
CYP3A4	CYP3A4*22	Decreased metabolism	Drug–drug interactions critical; ↑ exposure	Strong
CYP3A5	CYP3A5*3	Loss of function	Altered clearance (Asian populations)	Strong
ABCB1 (MDR1)	C3435T	Variable transport	CNS penetration; inconsistent associations	Limited
11β-HSD1	Various SNPs	Altered local activation	Tissue-specific GC effects	Emerging

Evidence levels in this table reflect the current body of published research on associations between genetic variants and glucocorticoid response variability. They do not imply clinical actionability as defined by CPIC, FDA, or other pharmacogenomic implementation consortia, for which none of the variants listed have yet achieved formal classification. Readers are referred to www.clinpgx.org and www.pharmgkb.org for updated actionability classifications.

GC, glucocorticoid; CPIC, Clinical Pharmacogenetics Implementation Consortium. ↑ = increased.

Receptor expression levels vary widely across tissues and individuals. Inflammatory conditions can downregulate GR expression, contributing to glucocorticoid resistance (6). Conversely, chronic glucocorticoid exposure triggers receptor downregulation and desensitization, reducing responsiveness over time (17). Post-translational modifications—including phosphorylation, acetylation, ubiquitination, and sumoylation—modulate receptor activity and stability (34).

Alternative splicing of NR3C1 generates receptor isoforms with distinct properties. The GRβ isoform, lacking the ligand-binding domain, acts as a dominant-negative inhibitor of GRα (the classic glucocorticoid receptor) and is elevated in glucocorticoid-resistant conditions. The GRα-A, GRα-B, GRα-C, and GRα-D isoforms, differing in N-terminal sequences, exhibit differential transcriptional activities and tissue distributions (34).

Cofactor availability influences glucocorticoid responsiveness. Coactivators (SRC-1, TIF2, and GRIP1) enhance transcriptional activity, while corepressors (NCoR and SMRT) suppress it. The balance between coactivators and corepressors varies across tissues and disease states, affecting glucocorticoid sensitivity (7). Chromatin accessibility, determined by histone modifications and DNA methylation, creates an epigenetic layer of variability in gene responsiveness (35).

Inflammatory mediators modify glucocorticoid action through multiple pathways. Pro-inflammatory cytokines (TNF-α and IL-1β) activate kinases (JNK and p38 MAPK) that phosphorylate GR, reducing its nuclear translocation and DNA-binding activity. Oxidative stress impairs receptor function through the direct oxidation of critical residues. These mechanisms contribute to acquired glucocorticoid resistance in chronic inflammatory diseases (36, 37).

### Disease-specific factors

Disease characteristics fundamentally influence glucocorticoid response. Inflammatory burden affects pharmacokinetics through altered protein binding, distribution, and clearance. Severe inflammation can reduce CBG levels, increasing free drug fractions and altering dose–response relationships. Conversely, inflammatory cytokines induce CYP3A4 suppression, potentially reducing metabolic clearance.

Disease activity modulates tissue responsiveness. In rheumatoid arthritis, synovial tissue GR expression and pro-inflammatory mediator levels predict treatment response. In asthma, airway inflammation severity correlates inversely with glucocorticoid sensitivity. Baseline disease severity often predicts both therapeutic response and required maintenance doses.

Comorbidities introduce additional complexity. Diabetes alters glucocorticoid metabolism and sensitivity. Obesity increases the volume of distribution and may reduce receptor sensitivity. Renal or hepatic impairment affects drug clearance and metabolite accumulation. Concurrent medications create interaction risks through metabolic and pharmacodynamic mechanisms (6).

## Clinical applications of precision glucocorticoid therapy

### Rheumatoid arthritis

Rheumatoid arthritis (RA) exemplifies the potential for biomarker-guided glucocorticoid therapy (38). Disease activity scores [DAS28, Clinical Disease Activity Index (CDAI), and Simplified Disease Activity Index (SDAI)] enable the quantitative assessment of treatment response and guide dose adjustments (39, 40). Inflammatory markers—C-reactive protein (CRP) and erythrocyte sedimentation rate (ESR)—correlate with disease activity and predict flare risk (41).

Multi-biomarker disease activity (MBDA) testing integrates 12 serum proteins into a composite score that predicts radiographic progression and treatment response (42). Patients with high MBDA scores require more aggressive glucocorticoid therapy and closer monitoring. Serial MBDA measurements enable early detection of inadequate response, allowing timely treatment escalation before irreversible joint damage occurs (42).

Pharmacogenomic testing identifies RA patients at high risk for glucocorticoid-related complications (43). ABCB1 polymorphisms predict both efficacy and toxicity in RA cohorts (44). The 3435C>T variant (rs1045642) is associated with reduced treatment response and increased infection risk (45). Patients carrying risk alleles may benefit from alternative therapies or enhanced monitoring protocols.

Anti-citrullinated protein antibodies (ACPAs) and rheumatoid factor (RF) not only aid diagnosis but also predict treatment response. Seronegative RA often requires different glucocorticoid strategies compared to seropositive disease. ACPA levels correlate with disease severity and may guide initial dose selection (46).

Imaging biomarkers complement clinical and laboratory assessments (47). Ultrasound detection of synovitis enables sensitive monitoring of subclinical disease activity (48). Power Doppler signal quantification predicts flare risk and guides glucocorticoid tapering (49). MRI synovitis and bone marrow edema scores correlate with treatment response and structural outcomes (50).

Treat-to-target strategies in RA incorporate biomarker-guided dose escalation and de-escalation (51, 52). Achieving remission (DAS28 <2.6) within 3–6 months predicts long-term joint preservation (53). Patients not reaching treatment targets despite adequate glucocorticoid doses require the addition of conventional or biologic DMARDs rather than further dose escalation (46, 54).

### Polymyalgia rheumatica

Polymyalgia rheumatica (PMR) represents one of the most common indications for long-term glucocorticoid therapy in adults over 50 years, with an incidence of 50–100 per 100,000 in this age group (55). The condition is characterized by bilateral shoulder and hip girdle pain and stiffness, elevated inflammatory markers, and dramatic response to glucocorticoid treatment. Despite clear diagnostic criteria and treatment guidelines, significant challenges persist in optimizing glucocorticoid dosing, minimizing cumulative exposure, and preventing relapses that affect 30%–50% of patients during tapering (56).

Current guidelines recommend initiating treatment with 12.5–25 mg/day prednisone equivalent, with most protocols converging on 15 mg/day as the standard starting dose (57, 58). However, substantial inter-individual variability exists in both the rapidity and completeness of response. Biomarker-guided approaches utilizing baseline ESR and CRP levels have shown promise for dose individualization, with patients exhibiting ESR > 70 mm/h or CRP > 50 mg/L potentially requiring higher initial doses to achieve complete symptom suppression (59). Pharmacogenetic studies, while limited in PMR-specific cohorts, suggest that NR3C1 variants associated with glucocorticoid resistance may contribute to the 10%–15% of patients who demonstrate inadequate response to standard dosing (60).

The temporal pattern of response itself provides prognostic information. Complete symptom resolution within 48–72 h of treatment initiation is highly characteristic of PMR and supports the diagnosis, while delayed or partial response should prompt reconsideration of alternative diagnoses such as late-onset rheumatoid arthritis, polymyositis, or occult malignancy (61). Quantitative assessment using patient-reported outcome measures and structured symptom diaries enables more objective response monitoring compared to traditional clinical assessment alone.

Glucocorticoid tapering in PMR remains controversial, with multiple competing protocols demonstrating different risk–benefit profiles (**Table 3**). Standard British Society for Rheumatology guidelines recommend a structured taper from 15 mg/day, reducing by 2.5 mg every 2–4 weeks until reaching 10 mg, followed by 1-mg decrements monthly, targeting discontinuation at 12–18 months (62). However, this approach is associated with relapse rates of 23%–35%, often necessitating dose escalation and prolonging total treatment duration.

**Table 3:** Glucocorticoid dosing strategies in polymyalgia rheumatica: evidence-based protocols and outcomes.

Protocol	Initial dose (mg/day)	Tapering schedule	Duration to discontinuation	Relapse rate (%)
Standard protocol (BSR/BHPR)	15	Reduce by 2.5 mg every 2–4 weeks until 10 mg, then 1 mg/month	12–18 months	23–35
EULAR recommendations	12.5–25	Individualized based on clinical response and biomarkers	≥12 months	20–30
Low-dose initial (LD-PMR)	10–12.5	Slow taper: 1 mg every 4–8 weeks after week 4	18–24 months	28–40
Rapid taper protocol	15–20	Aggressive: 2.5 mg every 2 weeks to 5 mg, then 1 mg/month	6–12 months	45–55
Biomarker-guided	15 (adjusted)	ESR/CRP-directed: reduce when normalized; hold if elevated	Variable (12–36 months)	15–25
Imaging-guided (PET/MRI)	12.5–15	Reduce based on vascular inflammation resolution	12–24 months	18–28
Methotrexate-sparing	15 + MTX 10 mg/week	Faster GC taper (50% reduction by 12 weeks)	9–15 months	12–20
Tocilizumab combination	15–20 + TCZ	Rapid GC discontinuation (within 24 weeks possible)	6–12 months	8–15

ESR, erythrocyte sedimentation rate; CRP, C-reactive protein; BSR/BHPR, British Society for Rheumatology/British Health Professionals in Rheumatology; EULAR, European League Against Rheumatism; LD-PMR, Low-Dose Polymyalgia Rheumatica.

This phenomenon reflects a fundamental paradox in PMR glucocorticoid therapy: inadequate initial dosing or overly aggressive early tapering, while minimizing short-term glucocorticoid exposure, frequently precipitates relapses requiring dose escalation and treatment reinitiation, ultimately prolonging total treatment duration and increasing cumulative glucocorticoid burden. Patients who achieve complete disease suppression with adequate initial dosing (typically 15–20 mg/day prednisone) often successfully taper and discontinue therapy within 12–18 months. Conversely, those started on insufficient doses (e.g., 10 mg/day) or subjected to rapid early taper frequently experience repeated flares necessitating years of low-dose maintenance therapy. This “less now, more later” paradox underscores the importance of individualized dose selection balancing immediate toxicity concerns against long-term cumulative exposure optimization.

Biomarker-guided tapering represents a precision medicine approach that adjusts dose reduction based on serial ESR and CRP measurements rather than fixed time intervals (63). This strategy holds taper when inflammatory markers begin to rise, even in the absence of overt symptoms, potentially identifying subclinical disease activity before clinical relapse occurs. Prospective studies demonstrate relapse rate reductions to 15%–25% using biomarker-directed protocols, although at the cost of longer total treatment duration and higher cumulative glucocorticoid exposure in some patients.

Emerging imaging modalities, particularly ^18^F-FDG PET/CT and whole-body MRI, reveal vascular and musculoskeletal inflammation in PMR that may persist despite clinical remission and normalized acute phase reactants (64, 65). Imaging-guided tapering protocols that defer dose reduction until the resolution of vascular uptake demonstrate promising results in pilot studies (65). However, cost-effectiveness analyses are needed to determine the optimal role of advanced imaging in routine PMR management.

The high relapse rate and prolonged treatment duration in PMR have motivated investigation of steroid-sparing agents to reduce cumulative glucocorticoid exposure and associated toxicity. Methotrexate, the most extensively studied adjunctive therapy, demonstrates modest and inconsistent efficacy in enabling faster glucocorticoid taper and reducing total cumulative dose. However, the absolute benefit remains limited, and current guidelines position methotrexate as optional rather than standard therapy. Patient selection for methotrexate co-therapy should consider baseline osteoporosis risk, diabetic status, and prior glucocorticoid-related complications (66).

Interleukin-6 inhibition with tocilizumab represents a paradigm shift in PMR treatment, targeting the key cytokine driver of disease pathogenesis (67, 68). Phase III trial data demonstrate that tocilizumab enables complete glucocorticoid discontinuation in 50%–60% of patients by 6 months, compared to <20% with glucocorticoid monotherapy (69, 70). While not yet approved specifically for PMR in most jurisdictions, tocilizumab therapy should be considered in patients with relapsing disease despite optimized glucocorticoid management, those experiencing significant toxicity, or individuals at particularly high risk for glucocorticoid-induced complications. Economic modeling suggests cost-effectiveness in these high-risk subgroups despite the substantial drug acquisition costs.

Systematic pharmacogenetic investigation in PMR remains limited, but extrapolation from other glucocorticoid-treated conditions suggests potentially actionable genetic variation. FKBP5 polymorphisms associated with hypothalamic–pituitary–adrenal axis sensitivity may identify patients at elevated risk for adrenal suppression during prolonged therapy, warranting earlier morning cortisol monitoring and a more gradual taper below 5 mg/day. VDR gene variants predictive of bone loss could inform more aggressive osteoporosis prevention strategies, potentially including early bisphosphonate initiation rather than delayed intervention based on dual-energy X-ray absorptiometry (DEXA) findings.

The integration of clinical, biomarker, imaging, and potentially genetic data into comprehensive prediction models for relapse risk, treatment duration, and complications represents an important frontier in PMR precision medicine. Such models could enable truly personalized treatment planning, moving beyond one-size-fits-all protocols toward individualized dose trajectories optimized for each patient’s unique risk profile.

### Inflammatory bowel disease

In inflammatory bowel disease (IBD), biomarkers enable precise assessment of mucosal inflammation independent of symptoms (71, 72). Fecal calprotectin strongly correlates with endoscopic disease activity in both Crohn’s disease and ulcerative colitis (73). Levels >250 μg/g indicate active inflammation requiring treatment intensification, while normalization (<50 μg/g) suggests mucosal healing (74, 75).

C-reactive protein monitoring in IBD is complicated by the fact that up to 25% of patients fail to mount a CRP response despite active inflammation (76). This subset, often carrying genetic variants affecting hepatic acute-phase response, requires alternative monitoring strategies (77). The combination of CRP with fecal calprotectin improves diagnostic accuracy and treatment guidance (78).

Therapeutic drug monitoring of concurrent immunosuppressives (thiopurines and anti-TNF agents) enables optimization of combination therapy with glucocorticoids (79, 80). Subtherapeutic drug levels predict glucocorticoid dependency and treatment failure (81). Dose optimization of steroid-sparing agents can facilitate glucocorticoid taper (82).

Pharmacogenomic markers predict both efficacy and adverse events in IBD. ABCB1 variants influence treatment response in ulcerative colitis, with the 3435TT genotype associated with glucocorticoid resistance (83, 84). Thiopurine S-methyltransferase (TPMT) polymorphisms, while primarily relevant to thiopurine metabolism, influence combination therapy strategies (85).

Steroid-refractory IBD, defined as failure to respond to prednisone 0.75 mg/kg/day within 4 weeks, occurs in 20%–30% of patients (86). Early identification through biomarker monitoring enables timely escalation to biologic therapies (80). Conversely, steroid-dependent IBD (inability to taper below prednisone 10 mg/day without relapse) affects 30%–40% of patients and necessitates alternative maintenance strategies (81).

Emerging biomarkers in IBD include serum and mucosal cytokine profiles that predict treatment response (87). IL-6, TNF-α, and interferon-γ levels correlate with disease severity and glucocorticoid sensitivity (88, 89). Transcriptomic signatures identify molecular subtypes with distinct therapeutic requirements (90).

### Asthma and chronic obstructive pulmonary disease

Asthma phenotyping guides glucocorticoid therapy selection and dosing (91). Type 2 high asthma, characterized by elevated eosinophils, fractional exhaled nitric oxide (FeNO), and IgE, exhibits excellent glucocorticoid responsiveness (92). Conversely, non-type 2 asthma (neutrophilic or paucigranulocytic) shows relative glucocorticoid resistance and may require alternative therapies (93, 94).

Blood eosinophil counts predict both inhaled and systemic glucocorticoid response (95). Eosinophils >300 cells/μL identify patients likely to benefit from inhaled corticosteroids, while counts <150 cells/μL suggest limited efficacy (96, 97). Serial eosinophil monitoring during treatment enables dose optimization and early detection of loss of control (98).

FeNO measurement provides real-time assessment of type 2 airway inflammation. Values >50 ppb indicate active eosinophilic inflammation amenable to glucocorticoid therapy (99). FeNO-guided treatment strategies reduce exacerbation rates by 20%–30% compared to symptom-based management (100, 101).

Pharmacogenomic predictors of glucocorticoid response in asthma include polymorphisms in GLCCI1 (glucocorticoid-induced transcript 1), which account for substantial variability in treatment response (102, 103). The rs37973 variant is associated with reduced lung function improvement following inhaled corticosteroids (102). FCER2 polymorphisms influence IgE regulation and treatment response in allergic asthma (104).

In chronic obstructive pulmonary disease (COPD), blood eosinophil counts similarly predict glucocorticoid response and exacerbation risk (95). Patients with eosinophils >300 cells/μL benefit from inhaled corticosteroid therapy, while those with counts <100 cells/μL show minimal benefit and increased pneumonia risk (105). This biomarker enables precision escalation and de-escalation of inhaled glucocorticoid therapy (106).

Sputum analysis provides direct assessment of airway inflammation (107). Sputum eosinophilia (>3%) predicts glucocorticoid responsiveness, while neutrophilic inflammation (>61%) suggests resistance (98). However, sputum induction is technically demanding and not widely available, limiting routine clinical application (91).

### Systemic lupus erythematosus

Systemic lupus erythematosus (SLE) presents unique challenges for glucocorticoid management due to disease heterogeneity and relapsing–remitting course (108). Disease activity indices [Systemic Lupus Erythematosus Disease Activity (SLEDAI) and British Isles Lupus Assessment Group (index) (BILAG)] enable standardized assessment of treatment response (109, 110). Serologic markers—anti-dsDNA antibodies and complement levels (C3 and C4)—correlate with disease activity and predict flares (111).

Rising anti-dsDNA titers or declining complement levels often precede clinical flares by weeks to months, enabling preemptive treatment intensification (112, 113). Conversely, normalization of these markers supports glucocorticoid tapering (108). However, 20%–30% of SLE patients maintain stable serologies despite active disease, requiring clinical correlation (114).

Urinary biomarkers enable sensitive monitoring of lupus nephritis (115). Urinary protein-to-creatinine ratio (UPCR) quantifies proteinuria and guides treatment intensity (116). Urinary biomarkers, including MCP-1, NGAL, and TWEAK, predict renal flares before creatinine elevation (117). Serial monitoring enables early detection of inadequate response to glucocorticoid therapy (111).

Interferon signature, measured through the expression of interferon-stimulated genes, predicts disease activity and treatment response in SLE. High interferon signature identifies patients requiring more intensive immunosuppression (108). However, interferon status remains relatively stable over time, limiting its utility for treatment monitoring (111).

Pharmacogenomic considerations in SLE include polymorphisms affecting both disease susceptibility and treatment response. FCGR polymorphisms influence immune complex clearance and disease severity (118). CYP2C19 variants affect metabolism of concurrent immunosuppressives (hydroxychloroquine and cyclophosphamide) and may influence combination therapy strategies (119).

## Implementation strategies for precision glucocorticoid therapy

### Pharmacogenomic testing infrastructure

Implementing pharmacogenomic-guided glucocorticoid therapy requires institutional infrastructure for genetic testing. Pre-emptive genotyping—testing patients before medication initiation—provides the most actionable results. Panel-based approaches testing multiple pharmacogenes simultaneously improve cost-effectiveness compared to single-gene testing (16).

Clinical Pharmacogenetics Implementation Consortium (CPIC) guidelines provide evidence-based recommendations for integrating genetic test results into prescribing decisions. While the CPIC has not yet published specific guidelines for glucocorticoids, existing frameworks for other drug classes provide implementation templates. Institutional pharmacogenomics programs require collaboration between clinical pharmacists, laboratory medicine, and informatics specialists (120).

Electronic health record (EHR) integration ensures that genetic test results inform prescribing at the point of care. Clinical decision support (CDS) alerts notify providers of high-risk genotypes and recommend dose adjustments or alternative therapies. Passive alerts (displaying information without requiring action) show better provider acceptance than interruptive alerts (121, 122).

Genetic counseling supports informed consent and result interpretation. Patients should understand testing limitations, the probabilistic nature of genetic predictions, and implications for family members. Standardized consent forms and educational materials facilitate efficient counseling.

### Biomarker monitoring protocols

Systematic biomarker monitoring requires institutional protocols specifying test selection, frequency, and interpretation. Disease-specific monitoring strategies are outlined in **Table 4**. General principles include baseline assessment before treatment initiation, frequent monitoring during dose titration (every 2–4 weeks), and less frequent monitoring during stable maintenance therapy (every 3–6 months).

**Table 4:** Disease-specific biomarkers informing glucocorticoid treatment decisions: from initiation to dose optimization.

Disease	Biomarker	Threshold/target	Clinical action	Implementation status	Primary role
COPD exacerbation	Blood eosinophils	≥300 cells/μL or ≥2%	Use systemic corticosteroids	**✓ Implemented (GOLD)**	**Initiation**
Asthma	FeNO	>50 ppb (adults)	ICS-responsive phenotype	Ready for practice	**Initiation**
Asthma	Blood eosinophils	≥300 cells/μL	Predict ICS response	Ready for practice	**Initiation**
Septic shock	IL-6	>40 pg/mL	Consider hydrocortisone	Research/validation	**Initiation**
ARDS	Inflammatory phenotype	Hyperinflammatory subtype	Corticosteroid benefit	Emerging evidence	**Initiation**
COVID-19 ARDS	CRP + hypoxemia	>150 mg/L + O_2_ requirement	Dexamethasone 6 mg daily	**✓ Implemented (WHO)**	**Initiation**
Rheumatoid arthritis	CRP, IL-6	Normalization	Guide tapering strategy	Emerging practice	**Monitor/taper**
SLE	Complement (C3, C4)	Low levels	Organ-specific dosing	Emerging practice	**Init/monitor**
IBD (Crohn’s)	Fecal calprotectin	<150 μg/g	Consider GC taper	Research phase	**Tapering**

COPD, chronic obstructive pulmonary disease; CRP, C-reactive protein; SLE, systemic lupus erythematosus; IBD, inflammatory bowel disease; ICS, inhaled corticosteroid(s). Bold text in the Primary Role column indicates the biomarker’s principal function within the treatment algorithm (initiation, monitoring, or tapering guidance).

Point-of-care testing for selected biomarkers (CRP and glucose) enables real-time treatment decisions. However, laboratory-based assays provide superior analytical performance for most biomarkers. Turnaround time optimization ensures that results are available for clinical decision-making.

Biomarker trajectories provide more information than isolated values. Worsening trends despite adequate therapy trigger treatment intensification, while improving trends support dose reduction. Statistical process control methods can identify significant deviations from expected patterns (123).

Multi-biomarker panels integrating complementary markers improve diagnostic accuracy. Commercial platforms (e.g., MBDA for rheumatoid arthritis) provide validated composite scores. Institution-specific panels can be designed based on disease characteristics and available testing capabilities (42).

### Economic considerations and health system implementation

The economic burden of glucocorticoid therapy extends beyond drug acquisition costs to encompass monitoring expenses, management of adverse effects, and productivity losses from treatment complications. Glucocorticoid-induced osteoporosis, diabetes, infections, and cardiovascular events impose substantial direct medical costs and indirect societal costs (**Table 5**). Annual healthcare expenditures attributable to glucocorticoid complications exceed $10 billion in the USA alone, with osteoporotic fractures, infections, and metabolic complications representing the largest cost drivers.

**Table 5:** Economic burden of glucocorticoid-related complications.

Complication category	Specific complication	Annual cost/event (USD)	Reference
Infections	Pneumonia requiring hospitalization	~$33,000/episode	(128)
	Sepsis/septic shock	$9,914/day (per diem)	(14)
Fractures	Hip fracture (total cost)	$1,743–$18,358/episode	(14)
	Vertebral compression fracture	$1,743–$18,358/episode	(14)
	Fracture-related surgery and rehabilitation	$1,743–$18,358/episode	(14)
Metabolic	New-onset diabetes (first year)	$8,000–$12,000	(129)
	Diabetes treatment (annual)	$9,000–$13,000/year	(129)
	Metabolic syndrome management	$5,411/year	(14)
Cardiovascular	Myocardial infarction	$26,472/event	(14)
	Stroke (acute + rehabilitation)	$35,000–$75,000	(130)
	Heart failure hospitalization	$15,000–$30,000	(131)
	Cardiovascular disease (annual)	$10,000–$25,000/year	(131)
Quality of life	QOL decrements (QALY loss)	$50,000–$100,000/QALY	(132)
	Healthcare utilization increase	+30–50% visits/year	(133)

All cost estimates are derived from US-based studies and reflect healthcare expenditures in USD at the time of original publication (14: 2009 USD; 131: 2021 USD; 130: 2008 USD). Costs should be interpreted as approximations given substantial variability across patient populations, disease severity, care settings, and payer types. Sepsis cost is reported as a per diem estimate ($9,914/day) rather than per episode. Fracture costs represent the full range across fracture types and severity levels. These estimates are not directly comparable across categories due to differences in methodology and reference year. Costs reported in non-2024 USD have not been adjusted for inflation.

QOL, quality of life; QALY, quality-adjusted life year.s

Pharmacogenomic testing presents upfront costs but may generate long-term savings through prevention of treatment failures and adverse events. Cost-effectiveness analyses demonstrate favorable incremental cost-effectiveness ratios for pharmacogenomic-guided therapy in patients requiring chronic glucocorticoid treatment (>6 months anticipated duration). For short-term therapy (<3 months), universal pharmacogenomic testing may not be cost-effective, although targeted testing in high-risk populations (elderly, multiple comorbidities, and prior adverse events) shows promise. The declining costs of genetic testing and increasing evidence for clinical utility continue to improve the economic case for precision approaches.

Biomarker monitoring costs vary widely depending on the specific tests employed. Basic inflammatory markers (ESR and CRP) are inexpensive and widely available, supporting their routine use in conditions such as polymyalgia rheumatica and rheumatoid arthritis. Advanced biomarkers including multi-biomarker disease activity panels, imaging studies (PET/CT and MRI), and specialized assays require careful cost–benefit analysis. Value-based implementation prioritizes biomarkers with demonstrated impact on clinical decision-making and patient outcomes rather than those providing marginal information gains.

Reimbursement for pharmacogenomic testing remains inconsistent across payers. Medicare covers selected tests with strong evidence (e.g., TPMT before thiopurine therapy), but coverage for glucocorticoid-related pharmacogenomics lags behind oncology and other therapeutic areas. Private insurers vary in their policies, with some requiring prior authorization and documented medical necessity. Advocacy for broader coverage requires demonstration of clinical utility, cost-effectiveness, and alignment with evidence-based guidelines.

Implementation science frameworks guide systematic adoption of precision glucocorticoid therapy. The RE-AIM framework (Reach, Effectiveness, Adoption, Implementation, Maintenance) provides a structured approach to program evaluation. Barriers include clinician knowledge gaps, workflow disruption, and institutional inertia. Facilitators include clinical champions, administrative support, and demonstrated value. Successful implementation requires addressing multiple stakeholder perspectives—patients, clinicians, administrators, and payers—each with distinct priorities and concerns (124).

### Glucocorticoid-induced osteoporosis: risk stratification and precision prevention

Glucocorticoid-induced osteoporosis (GIO) represents the most common form of secondary osteoporosis and a major source of morbidity in patients requiring chronic glucocorticoid therapy. Unlike postmenopausal osteoporosis, which develops over years to decades, GIO can manifest within months of treatment initiation, with bone loss most rapid during the first 6–12 months of therapy and fracture risk increasing within 3 months of starting glucocorticoids (125). The magnitude of risk varies substantially across individuals based on glucocorticoid dose, treatment duration, age, sex, baseline bone mineral density, and genetic factors, necessitating precision approaches to prevention and monitoring.

Glucocorticoids exert multifaceted deleterious effects on bone metabolism. Direct actions include decreased osteoblast differentiation and increased osteoblast and osteocyte apoptosis, reducing bone formation. Simultaneously, glucocorticoids increase osteoclast activity and prolong osteoclast lifespan, accelerating bone resorption. Indirect effects mediated through decreased intestinal calcium absorption, increased renal calcium excretion, secondary hyperparathyroidism, reduced sex hormone production, and myopathy further compound bone loss (126).

The dose–response relationship for GIO demonstrates a threshold effect, with fracture risk increasing significantly at doses ≥7.5 mg/day prednisone equivalent, although some studies suggest elevated risk even at lower doses when used chronically (127). Cumulative dose exposure correlates strongly with both bone mineral density decline and fracture incidence, emphasizing the importance of minimizing total glucocorticoid burden through effective tapering strategies and steroid-sparing agents where feasible.

Evidence-based guidelines from the American College of Rheumatology advocate for risk-stratified prevention strategies based on age, fracture history, glucocorticoid dose, and treatment duration (**Table 6**) (134). Patients can be broadly categorized into low, moderate, high, and very high risk groups, each warranting different intensities of monitoring and pharmacologic intervention.

**Table 6:** Glucocorticoid-induced osteoporosis: risk stratification, prevention strategies, and pharmacogenetic considerations.

Risk category	Risk factors	GC dose/duration threshold	Baseline assessment	Prevention strategy	Monitoring schedule	Pharmacogenetic markers
Low risk	Age <50, no RF, short-term GC	<7.5 mg/day, <3 months	Clinical assessment, Ca/Vit D status	Lifestyle, Ca 1,000–1,200 mg/day, Vit D 800–1,000 IU/day	DEXA if GC continues >3 months	VDR FokI (ff = better response to Vit D)
Moderate risk	Age 50–65, 1–2 RF, moderate GC	7.5–30 mg/day or any dose >3 months	DEXA, FRAX score, vertebral imaging if indicated	Ca/Vit D + consider bisphosphonate if T-score less than −1.5	DEXA every 2–3 years; annual height	COL1A1 Sp1 (SS = ↑ fracture risk)
High risk	Age >65, ≥3 RF, prior fracture	>30 mg/day or any dose if T-score less than −2.5	DEXA, lateral spine X-ray, biochemical markers (CTX, P1NP)	Bisphosphonate or denosumab; consider anabolic if very high risk	DEXA annually; height every 6 months	ESR1 XbaI/PvuII (associated with BMD response)
Very high risk	Prevalent vertebral fracture, T-score less than −3.0	Any GC dose/duration	DEXA, vertebral fracture assessment, bone turnover markers	Anabolic agent (teriparatide, romosozumab) preferred; transition to antiresorptive	DEXA 6–12 months; close clinical monitoring	LRP5 variants (Wnt pathway, bone formation)

Risk factors (RFs): postmenopausal status, family history of hip fracture, low body weight (<60 kg), current smoking, alcohol >3 units/day, previous fragility fracture, and secondary osteoporosis causes (rheumatoid arthritis, hypogonadism, malabsorption).

Low-risk patients—typically those under age 50 without prior fractures receiving short-term low-dose glucocorticoids—may be managed conservatively with calcium (1,000–1,200 mg/day) and vitamin D (800–1,000 IU/day) supplementation, lifestyle modifications including weight-bearing exercise and smoking cessation, and reassessment if glucocorticoid therapy extends beyond 3 months. Baseline DEXA scanning is not routinely recommended in this group unless additional risk factors emerge.

Moderate-risk individuals, including those aged 50–65 with one or two risk factors or receiving glucocorticoids ≥7.5 mg/day for more than 3 months, warrant baseline DEXA scanning and calculation of fracture risk using tools such as Fracture Risk Assessment Tool (FRAX). Pharmacologic intervention with bisphosphonates should be considered when T-score falls below −1.5 or FRAX-estimated 10-year major osteoporotic fracture risk exceeds 10%.

High-risk patients—those over 65 years, with three or more risk factors, or with prevalent vertebral fractures—require more intensive management. Baseline evaluation should include DEXA, lateral thoracolumbar spine radiographs or vertebral fracture assessment via DEXA, and biochemical markers of bone turnover (C-terminal telopeptide and procollagen type 1 N-terminal propeptide). Bisphosphonate therapy should be initiated at or shortly after glucocorticoid commencement rather than delayed until bone loss is documented.

Very high-risk individuals with existing vertebral fractures, T-scores less than −3.0, or multiple fragility fractures may benefit from anabolic therapy (teriparatide, abaloparatide, or romosozumab) rather than antiresorptive agents. Anabolic therapy builds new bone more rapidly than bisphosphonates, providing faster fracture risk reduction in this vulnerable population (135). Following anabolic therapy completion (typically 12–24 months), transition to antiresorptive therapy maintains the gains achieved.

Genetic variation contributes substantially to inter-individual differences in bone mineral density decline and fracture risk during glucocorticoid therapy. Vitamin D receptor (VDR) polymorphisms, particularly the FokI variant, influence vitamin D responsiveness and calcium absorption efficiency. Patients homozygous for the f allele (ff genotype) demonstrate superior bone mineral density response to vitamin D supplementation compared to FF homozygotes, suggesting potential utility for VDR genotyping to guide vitamin D dosing intensity (136).

Collagen type I alpha 1 (COL1A1) Sp1 binding site polymorphism associates with baseline bone mineral density and fracture risk in multiple populations. The SS genotype correlates with decreased bone mineral density and increased fracture susceptibility, identifying individuals who may benefit from more aggressive prevention strategies even before glucocorticoid exposure (137).

Estrogen receptor 1 (ESR1) variants influence bone response to estrogen signaling and have been associated with differential bone mineral density changes during glucocorticoid therapy, particularly in postmenopausal women (138, 139). The potential for pharmacogenetically guided selective estrogen receptor modulator therapy in high-risk genotypes warrants investigation.

LRP5 gene variants affecting the Wnt signaling pathway—critical for osteoblast function and bone formation—may identify individuals particularly vulnerable to glucocorticoid-mediated bone formation suppression (140, 141). Such patients might derive disproportionate benefit from anabolic therapy versus antiresorptive approaches.

Monitoring frequency should be risk-stratified, with higher-risk patients undergoing more intensive surveillance (134). For moderate-risk patients on chronic glucocorticoids, DEXA scanning every 2–3 years suffices for most. High-risk individuals warrant annual DEXA, with semi-annual height measurements to detect vertebral compression fractures. Very high-risk patients may benefit from 6–12-month DEXA intervals during the first years of therapy when bone loss is most rapid (142).

The optimal duration of osteoporosis prevention therapy during glucocorticoid treatment remains incompletely defined. Current evidence supports continuing bisphosphonate therapy throughout the duration of glucocorticoid exposure at doses ≥5 mg/day (142, 143). Following glucocorticoid discontinuation, some but not all bone loss is reversible, and continued osteoporosis treatment for 1–2 years post-discontinuation is often recommended, particularly in higher-risk patients (144). However, individualized approaches considering total bisphosphonate exposure duration (typically limiting to 5 years for oral bisphosphonates and 3 years for intravenous zoledronic acid) versus ongoing fracture risk are appropriate.

Economic analyses consistently demonstrate cost-effectiveness of calcium and vitamin D supplementation for all glucocorticoid-treated patients (134). Bisphosphonate therapy proves cost-effective in patients at moderate or higher risk, with incremental cost-effectiveness ratios well within accepted willingness-to-pay thresholds. Anabolic therapy, despite high acquisition costs, demonstrates cost-effectiveness specifically in very high-risk populations where fracture prevention rates substantially exceed those achieved with bisphosphonates (145, 146).

Implementation of systematic osteoporosis prevention in glucocorticoid-treated populations remains suboptimal, with studies indicating only 30%–50% of eligible patients receiving appropriate preventive therapy (147). Automated electronic health record alerts triggered by glucocorticoid prescriptions, standing orders for baseline DEXA in at-risk patients, and incorporation of fracture risk calculators into clinical decision support systems can improve prevention strategy adoption. Pharmacist-led osteoporosis prevention programs in specialty clinics demonstrate high success rates in ensuring appropriate supplementation and pharmacotherapy initiation.

Osteoporosis prevention must be integrated with broader precision glucocorticoid strategies rather than treated as an isolated concern. Genetic variants such as VDR polymorphisms may simultaneously influence both glucocorticoid responsiveness and bone loss susceptibility, suggesting potential for comprehensive pharmacogenetic panels guiding both dose selection and prevention strategies. Patients with NR3C1 variants conferring glucocorticoid resistance may require higher doses to achieve disease control but face correspondingly elevated osteoporosis risk, necessitating more aggressive bone protection.

The development of dual-purpose outcome measures that capture both disease activity control and treatment-related harms enables more nuanced benefit–risk optimization. For instance, in polymyalgia rheumatica or rheumatoid arthritis, the goal is not simply symptom suppression but rather achieving remission at the lowest cumulative glucocorticoid dose—a metric that simultaneously optimizes efficacy while minimizing osteoporosis and other toxicities. Such integrated approaches represent the essence of precision medicine: tailoring therapy to maximize benefit while minimizing harm based on individual patient characteristics.

Computerized CDS systems synthesize genetic, biomarker, and clinical data to generate treatment recommendations. Effective CDS integrates seamlessly into clinical workflow, provides actionable recommendations, and includes override capability for clinical judgment. Rule-based CDS uses explicit if–then logic to trigger alerts and recommendations, for example, “IF NR3C1 N363S genotype AND diabetes, THEN increase diabetes monitoring frequency.” Rule-based systems are transparent and easily validated but may not capture complex interactions (43).

Machine learning CDS identifies patterns in large datasets to predict outcomes and optimal treatments. These systems may achieve superior prediction accuracy but lack interpretability and require extensive training data. Hybrid approaches combining rule-based and machine learning methods may offer optimal performance (148).

CDS effectiveness depends on user acceptance and clinical integration. Alert fatigue from excessive notifications leads to override rates exceeding 90% in some systems. Optimization strategies include limiting alerts to high-risk situations, providing clear rationale and references, and enabling feedback loops for continuous improvement (43).

Cost-effectiveness analyses demonstrate favorable economic profiles for biomarker-guided glucocorticoid therapy across multiple conditions. Reduced treatment failures, decreased adverse events requiring intervention, and improved quality of life justify upfront testing costs. Incremental cost-effectiveness ratios (ICERs) typically fall below willingness-to-pay thresholds of $50,000–$100,000 per quality-adjusted life year (QALY) (129–131, 133, 149).

Budget impact analyses account for total healthcare costs including testing, monitoring, medications, and downstream consequences. Biomarker-guided strategies generally increase testing costs but reduce overall expenditure through improved outcomes. Time horizons of 1–5 years capture most economic benefits (133, 149).

Reimbursement for pharmacogenomic testing remains inconsistent across payers. Medicare covers selected tests with strong evidence (e.g., TPMT before thiopurines) but coverage for glucocorticoid pharmacogenomics is not established. Private payers variably cover genetic testing, often requiring prior authorization (150, 151).

## Future directions and research priorities

An important emerging development in glucocorticoid pharmacology is the advent of dissociative steroids—compounds designed to retain anti-inflammatory efficacy while reducing adverse effects by differentially modulating glucocorticoid receptor transactivation and transrepression pathways. Vamorolone (Agamree), the first approved dissociative steroid, received U.S. Food and Drug Administration (FDA) and European Medicines Agency (EMA) approval in 2023 for Duchenne muscular dystrophy. Acting as a selective glucocorticoid receptor modulator with additional mineralocorticoid receptor antagonism and membrane-stabilizing properties, vamorolone demonstrated similar efficacy to prednisone with a more favorable safety profile—notably without negative effects on bone biomarkers or linear growth—in the pivotal VISION-DMD randomized trial (152, 153). While currently approved only for Duchenne muscular dystrophy (DMD), dissociative steroids represent a conceptually important advance for the broader field of precision glucocorticoid therapy.

Nowhere is the gap between glucocorticoid use and its precision optimization more evident than in emergency medicine, where high-dose glucocorticoids are administered empirically across a wide range of acute conditions—anaphylaxis, status asthmaticus, adrenal crisis, and septic shock—often without consideration of individual pharmacogenomic or pharmacokinetic variability, underscoring the urgency of translating precision approaches beyond chronic disease management into acute care settings.

The field of precision glucocorticoid pharmacotherapy stands at an inflection point, with emerging technologies, expanding knowledge bases, and evolving clinical paradigms converging to enable truly individualized treatment approaches. While substantial progress has been made in identifying pharmacogenomic determinants, validating biomarker strategies, and developing implementation frameworks, critical gaps remain that must be addressed to realize the full potential of precision medicine in glucocorticoid therapy. This section outlines key research priorities, technological opportunities, and implementation challenges that will shape the next decade of progress in this field.

Artificial intelligence and machine learning represent transformative tools for integrating the multidimensional data required for precision glucocorticoid therapy. Current clinical decision-making struggles to synthesize pharmacogenomic profiles, pharmacokinetic parameters, biomarker trajectories, comorbidity patterns, and treatment history into cohesive individualized recommendations. Machine learning algorithms can identify complex, non-linear relationships among these variables that exceed human pattern recognition capabilities (154). Deep learning models trained on large-scale electronic health record datasets demonstrate promising performance in predicting glucocorticoid response, adverse events, and optimal dosing trajectories (148).

Natural language processing enables extraction of nuanced clinical information from unstructured clinical notes, radiology reports, and pathology findings that traditional structured data fields miss (155). For conditions such as polymyalgia rheumatica where symptom assessment remains subjective, natural language processing (NLP)-based analysis of clinical documentation may provide more sensitive response monitoring than conventional disease activity scores. The integration of these artificial intelligence (AI)-driven approaches into clinical decision support systems could provide real-time, patient-specific dosing recommendations that adapt dynamically as new clinical data accumulate.

Digital health technologies offer unprecedented opportunities for continuous, longitudinal monitoring beyond episodic clinic visits. Wearable devices measuring physical activity, sleep quality, heart rate variability, and other physiologic parameters can detect early signs of disease flare or glucocorticoid toxicity before they become clinically apparent (156). Patient-reported outcome measures collected via smartphone applications enable high-frequency symptom tracking with minimal patient burden (157). The integration of these digital biomarkers with traditional laboratory and imaging assessments creates a comprehensive, multidimensional view of treatment response.

Point-of-care pharmacogenetic testing represents a critical implementation enabler, potentially eliminating the weeks-long delay between sample collection and results availability that currently limits clinical utility (158). Rapid genotyping platforms capable of delivering actionable pharmacogenetic results within hours of sample collection would enable pre-treatment risk stratification even in acute settings. Coupling point-of-care genotyping with clinical decision support algorithms could facilitate same-day treatment individualization.

Multi-omics integration extending beyond single-gene pharmacogenomics to encompass transcriptomics, proteomics, metabolomics, and epigenomics promises more comprehensive characterization of individual variability (159). Polygenic risk scores incorporating multiple low-effect variants may improve prediction accuracy beyond single-gene testing (160). Metabolomic profiling could identify endogenous biomarkers of glucocorticoid exposure and effect, enabling therapeutic drug monitoring even for drugs without established concentration–effect relationships (161).

Despite growing evidence for pharmacogenomic associations in adult populations, pediatric precision glucocorticoid therapy remains understudied. Developmental changes in drug metabolism, receptor expression, and physiologic response create age-specific pharmacokinetic and pharmacodynamic profiles that differ fundamentally from adults (162). The long-term consequences of glucocorticoid exposure during critical developmental windows—affecting growth, neurodevelopment, and metabolic programming—necessitate particularly careful risk–benefit optimization in children (27). Establishing pediatric-specific pharmacogenomic associations, pharmacokinetic models, and clinical decision algorithms represents a high-priority research need.

Geriatric populations face distinct challenges, including altered pharmacokinetics from age-related physiologic changes, polypharmacy with attendant drug interactions, and elevated baseline risk for glucocorticoid complications including osteoporosis, diabetes, and cognitive impairment (163). Age-stratified studies examining whether pharmacogenomic associations observed in younger adults persist in elderly populations are lacking. The optimal balance between disease control and toxicity minimization may differ substantially in patients with limited life expectancy, requiring different therapeutic goals and decision frameworks.

Rare genetic variants identified through whole-genome and whole-exome sequencing studies may account for extreme phenotypes—patients with profound glucocorticoid resistance or exceptional susceptibility to adverse effects (164). While common variants identified through candidate gene and genome-wide association studies explain population-level variability, rare variants may drive individual extreme responses. Systematic collection and functional characterization of rare pharmacogenetic variants will enable personalized interpretation rather than population-based risk estimates.

Gene–gene and gene–drug–drug interactions add layers of complexity not captured by single-variant analysis. CYP3A4/5 inhibitors and inducers dramatically alter glucocorticoid exposure, but pharmacogenetic variants may modulate the magnitude of these interactions (165). ABCB1-mediated drug efflux affects multiple medications, creating potential for synergistic or antagonistic pharmacokinetic interactions. Comprehensive interaction databases and clinical decision support tools incorporating multi-drug, multi-gene interactions are needed.

Long-term comparative effectiveness research comparing precision-guided versus standard glucocorticoid therapy remains limited. While short-term studies demonstrate improved response rates and reduced adverse events with pharmacogenomic guidance, the impact on patient-centered outcomes—quality of life, disability, and mortality—over years to decades requires prospective longitudinal investigation (11). Cost-effectiveness from a societal perspective incorporating long-term complication prevention must be rigorously evaluated.

Translation of precision glucocorticoid therapy from research settings to routine clinical practice faces substantial implementation barriers. Clinician knowledge gaps regarding pharmacogenomic test interpretation and clinical application represent a primary obstacle (166). Current medical education provides limited training in genomic medicine, and practicing physicians express low confidence in applying genetic test results to treatment decisions. Systematic educational interventions, clinical decision support tools, and pharmacogenomic consultation services are needed to bridge this knowledge gap.

Workflow integration challenges impede adoption even when clinicians recognize the value of precision approaches. Ordering genetic tests, awaiting results, interpreting findings, and modifying treatment plans require time and effort in already constrained clinical workflows (167). Pre-emptive genotyping—testing patients before therapeutic need arises—addresses the timing barrier but requires institutional infrastructure and culture change. Electronic health record integration that surfaces genetic results automatically when glucocorticoid prescribing is contemplated could reduce implementation friction.

Health equity considerations are paramount, as precision medicine risks exacerbating existing disparities if implementation is concentrated in well-resourced healthcare systems serving advantaged populations (168). Pharmacogenomic studies have historically underrepresented non-European ancestry populations, limiting the applicability of findings to diverse patients (169). Allele frequencies, linkage disequilibrium patterns, and gene–environment interactions differ across populations, necessitating ancestry-specific validation studies. Active efforts to ensure equitable access to precision glucocorticoid therapy across socioeconomic, geographic, and racial/ethnic groups are essential.

Global implementation faces distinct challenges in resource-limited settings where even basic laboratory monitoring may be unavailable (170). Developing low-cost, point-of-care testing solutions and clinical algorithms that function without extensive laboratory infrastructure could democratize access to precision approaches. Telemedicine and mobile health platforms may enable specialist pharmacogenomic consultation in settings lacking local expertise.

Patient engagement and shared decision-making must be central to precision glucocorticoid therapy implementation. Genetic test results and probabilistic risk estimates require careful communication to support informed decisions without inducing anxiety or therapeutic nihilism (171). Decision aids translating complex pharmacogenomic information into patient-accessible formats facilitate meaningful participation in treatment planning. Understanding patient values, preferences, and priorities regarding trade-offs between efficacy and toxicity risk enables truly personalized treatment decisions.

Regulatory frameworks for pharmacogenomic testing and precision medicine applications continue to evolve. The FDA has issued guidance documents on pharmacogenetic testing and biomarker-guided therapy, but glucocorticoid-specific guidance remains limited (172). As evidence accumulates, regulatory endorsement through drug labeling updates and clinical practice guideline incorporation will accelerate adoption. Pharmacogenomic consortia including the CPIC provide evidence-based recommendations, but glucocorticoid-related guidelines are underdeveloped compared to other therapeutic areas.

Reimbursement policies lag behind clinical evidence, with inconsistent coverage for pharmacogenomic testing across payers creating access barriers (120). Demonstrating cost-effectiveness from payer perspectives—including both short-term medication costs and long-term complication prevention—is essential for securing coverage. Value-based care models that reward outcomes rather than service volume may better align incentives with precision medicine goals.

Data sharing and registry development enable the large-scale studies required to validate rare variant associations, characterize diverse populations, and assess long-term outcomes (173). Federated data networks preserving patient privacy while enabling multi-site analysis represent promising infrastructure. Standardized phenotyping protocols ensure data compatibility across studies. International collaboration is essential to achieve the sample sizes necessary for definitive answers to key research questions.

**Figure 3:**
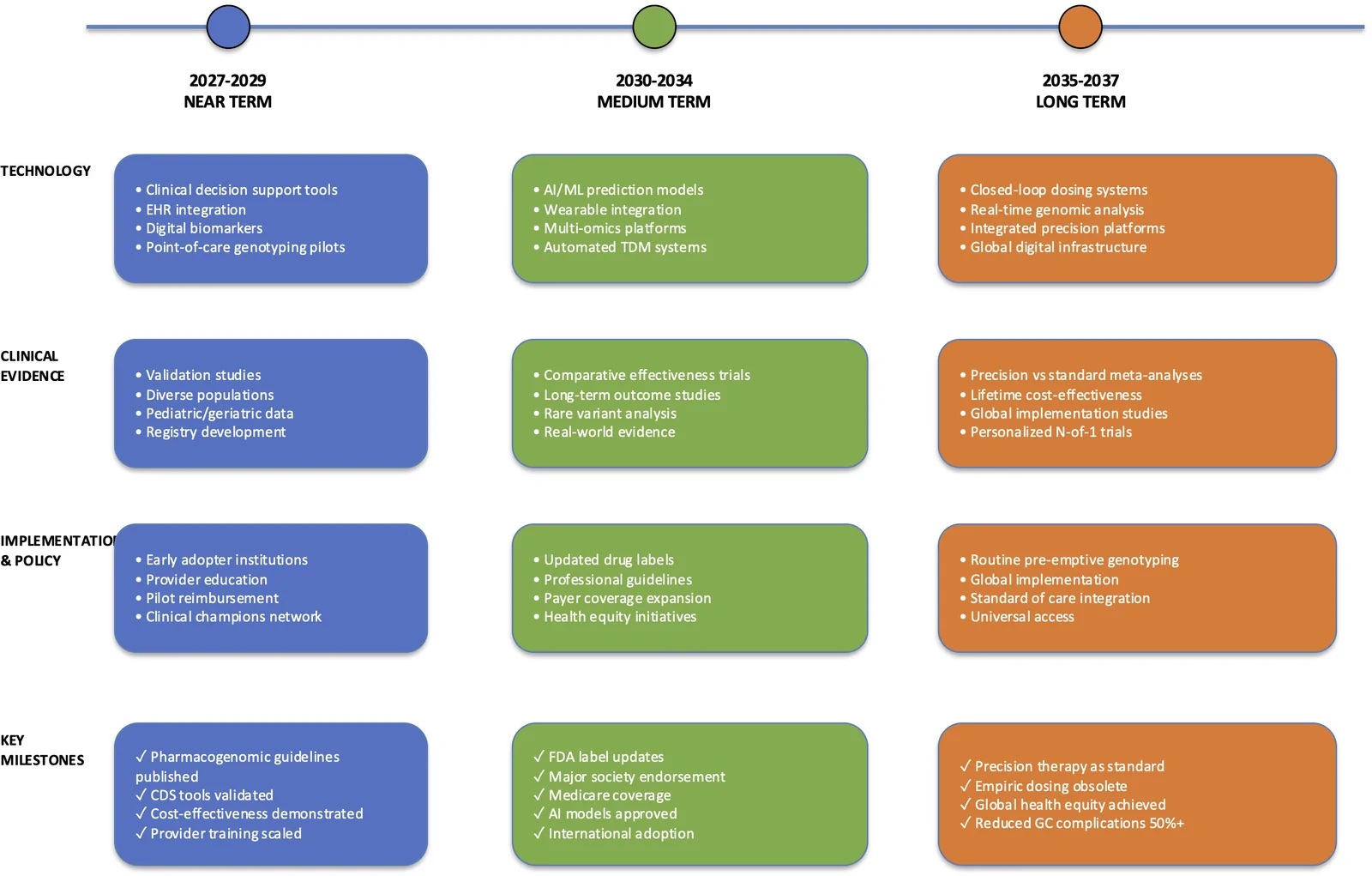
Roadmap for precision glucocorticoid therapy: 2027–2037. Strategic implementation roadmap illustrating the projected evolution of precision glucocorticoid therapy across three timeframes and parallel development domains. Near-term priorities (2027–2029, blue) focus on validation studies in diverse populations, development of clinical decision support tools with electronic health record integration, expansion of digital biomarker capabilities, initiation of point-of-care genotyping pilot programs, and establishment of early adopter institutional implementations with provider education initiatives. Medium-term goals (2030–2035, green) encompass deployment of artificial intelligence and machine learning prediction models, integration of wearable device monitoring, advancement of multi-omics profiling platforms, conduct of comparative effectiveness trials demonstrating outcomes superiority of precision approaches, achievement of regulatory drug label updates incorporating pharmacogenomic guidance, integration into professional society clinical practice guidelines, and expansion of payer reimbursement coverage. Long-term vision (2036–2037, orange) includes implementation of routine pre-emptive genotyping for at-risk populations, development of closed-loop personalized dosing systems integrating real-time data streams, achievement of global implementation extending precision approaches to resource-limited settings, transition of precision glucocorticoid therapy from specialized to standard clinical practice, and demonstration of ≥50% reduction in glucocorticoid-related complications at the population level. The three parallel tracks represent technological infrastructure advancement (top), clinical evidence generation (middle), and implementation science with policy evolution (bottom). Key milestones marked for each phase include publication of pharmacogenomic guidelines, FDA regulatory approvals, major professional society endorsements, Medicare coverage decisions, and achievement of health equity benchmarks. The timeline represents an aspirational trajectory contingent on sustained research funding, successful validation studies, and coordinated multi-stakeholder implementation efforts.

Achieving widespread implementation of precision glucocorticoid therapy will require coordinated efforts across multiple stakeholders and domains (**Figure 3**). Near-term priorities (2027–2029) include completing validation studies for established pharmacogenomic associations in diverse populations, developing clinical decision support tools integrating genetic and non-genetic data, and expanding educational initiatives for healthcare providers. Health systems with existing pharmacogenomic programs should serve as early adopters, generating real-world evidence and refining implementation strategies.

Medium-term goals (2030–2034) encompass developing and validating AI-driven prediction models, establishing point-of-care testing infrastructure, and conducting comparative effectiveness trials of precision versus standard approaches. Regulatory submissions for updated drug labeling incorporating pharmacogenomic guidance should be pursued. Professional society guidelines should integrate precision medicine recommendations. Reimbursement frameworks supporting pharmacogenomic testing should be established.

Long-term vision (2035–2037) includes routine pre-emptive genotyping for patients likely to require glucocorticoid therapy, comprehensive multi-omics profiling enabling systems-level treatment individualization, and global implementation extending precision approaches to resource-limited settings. The integration of genomic, digital health, and artificial intelligence technologies will enable closed-loop personalized dosing systems that continuously adapt therapy to individual patient trajectories. 

The transformation of glucocorticoid therapy from empirical to precision-based represents one component of the broader precision medicine revolution. Lessons learned—successful implementation strategies, overcoming barriers, and validated methodologies—will inform precision approaches to other complex, widely used medication classes. The ultimate goal is not simply optimizing glucocorticoid therapy in isolation but demonstrating a generalizable paradigm for evidence-based treatment individualization applicable across pharmacotherapy.

## Conclusions

Precision glucocorticoid pharmacotherapy represents a paradigm shift from empirical, one-size-fits-all dosing to individualized treatment based on patient-specific characteristics. The integration of pharmacogenomic testing, disease-specific biomarker monitoring, pharmacokinetic modeling, and clinical decision support enables optimization of therapeutic outcomes while minimizing toxicity.

The evidence base supporting precision approaches continues to strengthen across therapeutic areas. Pharmacogenomic variants in NR3C1, ABCB1, and CYP3A4/5 significantly influence glucocorticoid response and adverse effects. Disease-specific biomarkers—including inflammatory markers, autoantibodies, and imaging findings—enable real-time treatment monitoring and individualized dose adjustments.

Economic analyses consistently demonstrate favorable cost-effectiveness for biomarker-guided strategies. Reduced treatment failures, decreased glucocorticoid-related complications, and improved quality of life offset upfront testing costs. Budget impact models support institutional adoption from a financial perspective.

Successful implementation requires systematic attention to infrastructure, education, and workflow integration. Institutional pharmacogenomics programs, biomarker monitoring protocols, clinical decision support systems, and interdisciplinary collaboration provide essential foundations. Provider and patient education ensures appropriate test utilization and result interpretation.

Future developments—including advanced omics integration, artificial intelligence, wearable sensors, and gene editing—promise to further enhance precision glucocorticoid therapy. However, addressing evidence gaps, implementation barriers, and ethical considerations remains essential for equitable access to precision medicine benefits.

The transition from empirical to precision-based glucocorticoid therapy is already underway. As our understanding deepens and implementation tools become more accessible, precision approaches will increasingly become the standard of care. The ultimate goal—maximizing therapeutic benefit while minimizing harm for each patient—is achievable through systematic integration of pharmacogenomic, biomarker, and clinical data into treatment decision-making.
